# A Macrophage Membrane–Polymer Hybrid Biomimetic Nanoplatform for Therapeutic Delivery of Somatostatin Peptide to Chronic Pancreatitis

**DOI:** 10.3390/pharmaceutics14112341

**Published:** 2022-10-30

**Authors:** Fang Wang, Yu Deng, Luying Yu, Ao Zhou, Jieting Wang, Jingyan Jia, Ning Li, Fadian Ding, Wei Lian, Qicai Liu, Yu Yang, Xinhua Lin

**Affiliations:** 1 College of Pharmaceutical Science, Zhejiang University of Technology, Hangzhou 310014, China; 2 Key Laboratory of Nanomedical Technology (Education Department of Fujian Province), Nanomedical Technology Research Institute, School of Pharmacy, Fujian Medical University, Fuzhou 350122, China; 3 Center for Reproductive Medicine, 1st Affiliated Hospital, Fujian Medical University, 20 Chazhong Road, Fuzhou 350005, China; 4 Department of Hepatopancreatobiliary Surgery, The Third Affiliated Hospital of Soochow University, Juqian Road 185, Changzhou 213000, China

**Keywords:** peptide delivery, chronic pancreatitis, somatostatin, macrophage membrane, biomimetic nanoparticles

## Abstract

The clinical translation of therapeutic peptides is generally challenged by multiple issues involving absorption, distribution, metabolism and excretion. In this study, a macrophage membrane-coated poly(lactic-co-glycolic acid) (PLGA) nanodelivery system was developed to enhance the bioavailability of the somatostatin (SST) peptide, which faces the hurdles of short half-life and potential side effects in the treatment of chronic pancreatitis. Using a facile nanoprecipitation strategy, SST was loaded in the nanoparticles with an encapsulation efficiency (EE) and a loading efficiency (LE) of 73.68 ± 3.56% and 1.47 ± 0.07%, respectively. The final formulation of SST-loaded nanoparticles with the camouflage of macrophage membrane (MP-SST) showed a mean diameter of 151 ± 4 nm and an average zeta potential of −29.6 ± 0.3 mV, which were stable long term during storage. With an above 90% cell viability, a hemolysis level of about 2% (<5%) and a preference for being ingested by activated endothelial cells compared to macrophages, the membrane–polymer hybrid nanoparticle showed biocompatibility and targeting capability in vitro. After being intravenously administered to mice with chronic pancreatitis, the MP-SST increased the content of SST in the serum (123.6 ± 13.6 pg/mL) and pancreas (1144.9 ± 206.2 pg/g) compared to the treatment of (Dulbecco’s phosphate-buffered saline) DPBS (61.7 ± 6.0 pg/mL in serum and 740.2 ± 172.4 pg/g in the pancreas). The recovery of SST by MP-SST downregulated the expressions of chronic pancreatitis-related factors and alleviated the histologic severity of the pancreas to the greatest extent compared to other treatment groups. This augmentation of SST therapeutic effects demonstrated the superiority of integrating the synthetic polymer with biological membranes in the design of nanoplatforms for advanced and smart peptide delivery. Other peptides like SST can also be delivered via the membrane–polymer hybrid nanosystem for the treatment of diseases, broadening and promoting the potential clinical applications of peptides as therapeutics.

## 1. Introduction

Chronic pancreatitis is characterized by ongoing inflammation which results in pathophysiology including acinar cell injury, duct dysfunction, fibrosis and neuroimmune crosstalk. Complications of the disease comprise abdominal pain, diabetes mellitus, exocrine pancreatic insufficiency, metabolic bone disease and even pancreatic cancer [1,2]. Since there are no medical therapies to interrupt or reverse the progression of the disease, the screening for and dealing with related complications are the major clinical managements for chronic pancreatitis [3,4]. SST, a cyclic peptide containing 14 amino acids, could inhibit the secretion of hormones, such as growth hormone, insulin, glucagon, gastrin, gastric acid and pancreatic enzyme. The therapeutic effects of SST were found in patients with acute or chronic pancreatitis [5,6,7,8]. However, with its peptide nature, SST can be easily degraded by the peptidase in plasma/tissue and suffers from a very short half-life (<1~3 min), which limits its clinical translation [9]. To improve the bioavailability, SST analogues such as octreotide acetate, lanreotide and pasireotide have been developed to obtain t_1/2_ ranging from 2 h to 600 h [10,11,12]. Nevertheless, except for the ability of pasireotide to bind to four SST receptors, most of the analogues only bind to the two of the five somatostatin receptor subtypes which attenuate their efficacies [13,14]. It was, therefore, important to develop strategies to prolong the bioactivity of SST in its native form in vivo. Given the wide distribution of SST receptors throughout the central nervous system and periphery [9], new formulations of SST capable of tissue targeting may be necessary to avoid or alleviate potential side effects. 

Nanotechnology, with its ability to improve the performance parameters of drugs, such as stability, circulation time and tissue/cell targeting, holds promise for advancing clinical translation of drugs with poor bioavailability [15,16,17]. The biological barriers of therapeutic peptides could be overcome via nanoparticle-based delivery approaches which have promoted the translation of pre-clinical peptides from the bench to the bedside [18,19,20,21]. SST and its analogues in lipidic, magnetic and polymeric nano/micro-particle formulations have been developed for therapies of acromegaly, tumor and gastroenterological conditions [22,23,24,25]. However, fewer nanoformulations enabling targeting and sustained release of SST for the treatment of chronic pancreatitis have been created. With biodegradability and biocompatibility, the PLGA nanoparticles are favorable carriers for long-term and sustained release of drugs in vivo to prolong the effects of therapeutics which have been approved by the FDA [26,27,28]. Surface engineering through linkage/conjugation of the polyethylene glycol (PEG), targeting ligands or antibodies can endow PLGA nanoparticles with more sophisticated functions [29,30,31,32]. However, since each chemical modification may offer only one kind of property to nanoparticles, the functional improvements via these bottom-up fabrication strategies are limited, which may result in restricted augmentation of final delivery efficiency compared to nanoparticles without modification [33,34,35].

As a top-down strategy to replicate the collective functions of biological systems, the cell membrane coating technology opened an avenue for nanoparticles to mimic cells with their internal properties such as targeting, biocompatibility, long circulation time and immunomodulation [36,37,38,39]. Macrophages are innate characters to respond to inflammation which can inherently migrate to the sites of injuries [40]. Containing the ligands of chemokines and adhesion molecules, the cell membranes play important roles in the targeting mobility of macrophages [41,42,43]. Through transferring the macrophage membranes to the surface of nanoparticles, the targeting capability of these nanoformulations could be enhanced, which has been demonstrated in the treatment of cancer and cardiovascular disorders, including vascular inflammation and atherosclerosis [44,45,46]. In addition to the targeting performance, the macrophage membranes could be leveraged for the detoxification and sequestration of pro-inflammatory cytokines due to the specific interaction of the membranes with inflammatory factors and endotoxins [47,48]. All these bioactivities of macrophage membranes imply their great potentials to augment the functions of nanoparticles. 

**Figure 1:**
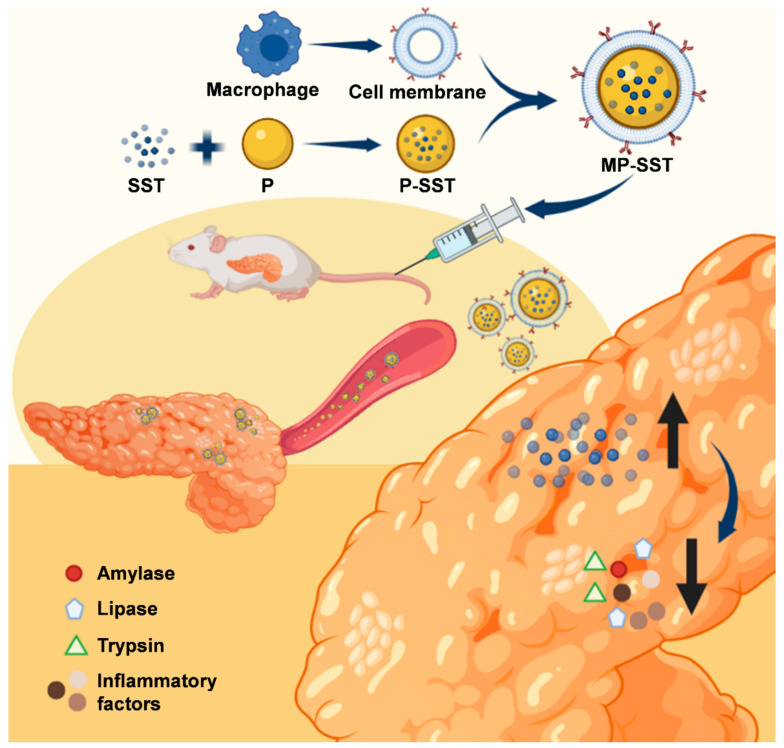
The macrophage membrane-coated PLGA nanoparticles loaded with SST peptide for the treatment of chronic pancreatitis.

Taking advantage of the biodegradable PLGA polymer and the natural macrophage membrane materials, this study aimed to develop a biohybrid nanosystem composed of SST-loaded polymer nanoparticle and a biosurface of macrophage membrane for the treatment of chronic pancreatitis (Figure 1). Compared with previous SST-loaded bare nanoparticles, the macrophage membrane could endow the PLGA nanoparticles with properties such as stability, biocompatibility and inflammatory targeting in addition to sustained release in vivo, which has been demonstrated previously [44,45,46]. Since almost no membrane-coated SST-containing nanoparticles have been developed yet, the study may provide a novel nanoformulation for SST therapeutic delivery. The capabilities of the MP-SST to improve the content of SST in pancreas and the effects of these biomimetic nanoparticles to augment the therapeutic functions of peptides were investigated. In addition to offering a new formulation of SST for the treatment of chronic pancreatitis, the MP-SST system demonstrated the promise of the hybridization of synthetic and biomembrane materials to improve the bioavailability and efficacies of peptide drugs which usually confront obstacles such as poor pharmacokinetics and unfavorable biodistribution for clinical applications [49,50].

## 2. Materials and Methods

### 2.1. Mateirals

PLGA (50:50, RG502H) and diethyldithiocarbamate (DDC) were obtained from the Sigma-Aldrich (Shanghai, China). Somatostatin (SST) was bought from the GL Biochem (Shanghai) Ltd. (China), and 0.9% sodium chloride solution for injection was obtained from the Hai Wang Fu Yao pharmacological industry of Fuzhou (Fujian, China). Endothelial cell medium (ECM) for culturing of endothelial cells was obtained from the UBIGENE (Guangzhou, China). Tryzol reagent for cell lysis was obtained from the Solarbio (Beijing, China), and 4% of paraformaldehyde (PFA) for fixation of cells and tissues was bought from the BOSTER Biological Technology co. ltd. (Wuhan, China). Dulbecco’s phosphate buffer saline (DPBS) and DMEM with high glucose for cell culture of macrophages were purchased from the Hyclone (Logan, UT, USA). BCA kit for quantification of proteins, dimethyl sulfoxide (DMSO), 4′,6-diamidino-2-phenylindole (DAPI), sodium dodecyl sulfate-polyacrylamide gel electrophoresis (SDS-PAGE) kit for protein gel electrophoresis and the loading buffer of proteins were bought from the Beyotime Biotechnology (Beijing, China). The enzyme linked immunosorbent assay (ELISA) kits were purchased from Jiangsu Meimian Industrial Co., Ltd. (Jiangsu, China). All solutions and materials for hematoxylin and eosin (HE) staining and Masson’s trichrome staining were obtained from Servicebio (Wuhan, China). Other chemicals or reagents and equipment used are specified in each experiment. 

### 2.2. Fabrication of Nanoparticles

The PLGA nanoparticles were fabricated via a previously reported facile nanoprecipitation method with some modification [51,52]. Specifically, 10 mg of PLGA was dissolved in DMSO and added to 10 mL of pure water dropwise. After the stirring under an ice bath for 1 h, the nanoparticle solution was transferred to the ultrafiltration tube with 10 kD MWCO (Millipore, MA, USA) and was purified by centrifugation under 3000× *g* for 15 min. The purification step was repeated 3 times following which the final nanoparticles were obtained and stored at 4 °C. To load the peptide, SST and PLGA were co-dissolved in DMSO at different weight ratios (1:10, 1:20, 1:50 and 1:100) of SST/PLGA and the process following to fabricate the P-SST nanoparticles was the same as the fabrication of PLGA nanoparticles. To isolate the plasma cell membrane, 5~30 million macrophages were needed. The RAW264.7 macrophages were obtained from the National Collection of Authenticated Cell Cultures of China (Shanghai, China) and were cultured in the DMEM cell culture medium supplemented with 10% FBS and 1% PS in the incubator (Esco CelCulture, CCL-170B-8). After the collection of the cells, the cell membrane was extracted by a kit (Invent Biotechnologies, Inc., Plymouth, MN, USA) and utilized for coating onto the PLGA nanoparticles via through a sonication strategy or otherwise stored in the DPBS under −20 °C [53,54]. To coat the nanoparticles, the cell membrane and PLGA nanoparticles at PLGA/cell membrane protein weight ratio of 5:1 were mixed and sonicated under an ice bath for 10 min. To exclude the extra cell membrane vesicles, the nanoparticle solution was centrifuged under 4500× *g* for 15 min.

### 2.3. Characterization of Nanoparticles

The diameters and zeta potentials of nanoparticles with concentration of 1 mg/mL were analyzed by dynamic light scattering (DLS) using LiteSizer (Anton Paar, Shanghai, China) at indicated times after the storage in water. The morphology of nanoparticles was observed with a FEI 200 kV transmission electron microscopy (TEM) (Tecnai Gl2, America) after negative staining with 1% uranyl acetate on a 400-mesh carbon-coated copper grid (Beijing Zhongjingkeyi Technology Co., Ltd., Beijing, China) [55].

### 2.4. Encapsulation and Loading Efficiency

The amount of SST in the nanoparticles was analyzed by a spectroscopic method [56]. Specifically, after dissolving of the lyophilized nanoparticles in the DMSO, the absorbance of solution at the maximum peak of SST ultraviolet–visible (UV-VIS) spectrum (284 nm) was analyzed and the content of SST was calculated according to the standard curve of the pure SST solution in DMSO. The drug encapsulation efficiency (EE) and loading efficiency (LE) were calculated as follows:

(1) $$\mathrm{LE}\;(\%)=\frac{W_{\mathrm{SST}}}{W_{\mathrm{PLGA}}+W_{\mathrm{SST}}}\times 100$$

(2) $$\mathrm{EE}\;(\%)=\frac{W_{\mathrm{SST}}}{W_{\mathrm{input}}}\times 100$$

where W_SST_ is the weight of SST loaded in the nanoparticles, W_PLGA_ is the weight of polymer in the formulation and W_input_ is the weight of SST added. 

### 2.5. Cumulative Release

SST released from PLGA nanoparticles was analyzed using a dialysis method. Briefly, 1 mL of MP-SST nanoparticle solutions (containing 100 μg SST) were added to dialysis bag (MWCO: 10,000 Da) which was then immersed in 5 mL of DPBS (pH 7.4) at 37 °C on a constant temperature oscillator (SHA-C, Changzhou Guohua electrics Co., Ltd., Changzhou, China) with shaking speed of 120 rpm; 500 μL of release medium was collected for analysis at different time intervals and replaced with an equivalent volume of fresh DPBS at 37 °C. The collected samples were lyophilized and dissolved in DMSO. As shown previously in Section 2.4, the contents of SST in the dissolved solutions were analyzed via the detection of absorbance at 284 nm.

### 2.6. SDS-PAGE

To analyze the protein composition of the membrane coated nanoparticles, the SDS-PAGE analysis was performed. Briefly, the separation and concentration gel were made according to the protocol of the kits. After the isolation and denaturation of the cell membrane related materials, the samples including the cell lysis, cell membrane vesicles and the PLGA nanoparticles with or without membrane coating were mixed with the loading buffer and loaded to the gel with total amount of 30 μg proteins. The voltage for samples in the concentration gel was 80 V for 30~40 min and 120 V for 50~60 min in the separation gel. After the Coomassie blue staining for 30 min, the gel was washed by deionized water and imaged under the BIO-RAD, Universal Hood Ⅱ. 

### 2.7. Biocompatability In Vitro

To investigate the cytotoxicity of nanoparticles, the human umbilical vein endothelial cells (HUVECs) obtained from ScienceCell (San Diego, CA, USA) were incubated in the 96-well plates with the cell density of 20,000/well using the ECM supplemented with 10% FBS, 1% PS and 1% ECGS. The PLGA nanoparticles without (P) or with membrane coating (MP) were added to the HUVECs with final concentration of 0.2 mg/mL and incubated for 48 h. After washed by DPBS for three times, the culture medium was replaced with ECM without FBS containing 10% cell counting kit-8 (CCK8) (Beyotime Biotechnology, Beijing, China) for 4 h of incubation. The absorbance of cells was analyzed at 450 nm. 

The hemocompatibility was studied by direct contact of nanoparticles with the blood extracted from ICR mice. After the addition of normal saline to equal volume of blood, the samples were centrifuged under 2500 rpm for 15 min to get the sedimentation of red blood cells. The P and MP nanoparticles in saline with concentration of 0.5 mg/mL or 1 mg/mL were added into the suspension of 2% red blood cells in saline and the mixed solution was incubated under the 37 °C for 1 h. After the centrifugation under 3000 rpm for 5 min, the absorbance of the supernatants was analyzed at 540 nm by a microplate reader (1510, Thermo Fisher Scientific, Waltham, MA, USA) to investigate the content of hemoglobin released from erythrocytes. The red blood cells treated with pure water and normal saline without nanoparticles were utilized as positive and negative controls, respectively. The hemolytic rate was calculated by:

$$\mathrm{Hemolysis}\;(\%)=\frac{{\mathrm{OD}}_{a}-{\;\mathrm{OD}}_{b}}{{\mathrm{OD}}_{c}-{\;\mathrm{OD}}_{b}}\times 100$$

where OD_a_, OD_b_ and OD_c_ represented the absorbances of the samples, the negative and positive control, respectively.

### 2.8. Cellular Uptake

To investigate the targeting and macrophage escape ability of nanoparticles, the HUVECs and RAW246.7 macrophages were incubated in the 96-well plates with cell density of 10,000 cells/well. The HUVECs were activated with 10 ng/mL tumor necrosis factor-α (TNF-α) for 6 h before the addition of the 1,1′-dioctadecyl-3,3,3′,3′-tetramethylindocarbocyanine perchlorate (DiI) (ThermoFisher Scientific, Waltham, MA, USA)-labeled nanoparticles with concentration of 0.1 mg/mL. After the incubation for 30 min in the incubator under 37 °C and 5% CO_2_, the cells were washed by the DPBS three times, fixed by 4% PFA for 30 min, stained by 1 μg/mL DAPI for 10 min and imaged under a confocal microscope (Leica SP5, Germany) with the excitation at 549 nm and the emission at 565 nm. 

### 2.9. The Administration of Nanoparticles to Mice with Chronic Pancreatitis

All the animal care and experimental protocols were performed in accordance with protocols reviewed and approved by the Animal Ethical and Experimental Committee of the Fujian Medical University. The male ICR mice (6~8 weeks of age, about 20 g) were obtained from the Experimental Animal Center of Fujian Medical University. The chronic pancreatitis model of mice was established as previously reported [57,58]. Briefly, 100 mg/mL DDC was intraperitoneally injected with the dose of 500 mg/kg twice a week for 4 weeks. After 2 weeks of DDC injection, the animals were weighed and then randomly assigned to intravenously receive: 100 μL of DPBS (*n* = 5), free SST (*n* = 6), SST-loaded PLGA nanoparticles (P-SST) (*n* = 6) and membrane-coated P-SST nanoparticles (MP-SST) (*n* = 7) at the dose of 250 μg/kg SST once a week. The healthy mice without DDC induction and therapeutic treatment (*n* = 6) were utilized as positive control. After four treatments, the rats were euthanized and the blood and organs were extracted at 1 week post final administration. 

### 2.10. ELISA

The concentrations of SST, lipase, amylase, trypsin, TNF-α, interleukin-1β (IL-1β) and interleukin-6 (IL-6) from the serum and the pancreas samples were analyzed using the ELISA kits according to the manufacturer’s instructions. The plates were incubated for 60 min at 37 °C after the adding of samples and enzyme solutions successively. The chromogen solution was added to react for 15 min under 37 °C and the absorbance of wells at 450 nm within 15 min was analyzed following the final addition of termination solution. 

### 2.11. Hematoxylin–Eosin and Masson’s Trichrome Staining

The pancreases extracted from the mice were immersion-fixed in 4% PFA for at least 12 h. Then, the cross sections were prepared and dewaxed (Xylene I for 20 min; Xylene II for 20 min; 100% ethanol I for 5 min; 100% ethanol II for 5 min; 75% ethanol for 5 min; rinsing with tap water). After being stained with hematoxylin solution for 3–5 min, Hematoxylin Differentiation solution, Hematoxylin Scott Tap Bluing, 85% ethanol for 5 min, 95% ethanol for 5 min and eosin dye for 5 min successively, the sections were dehydrated and observed by an upright optical microscope (Nikon Eclipse E100, Tokyo, Japan) and an imaging system (Nikon, Nikon DS-U3). To stain the sections with Masson’s trichrome, the sections were dewaxed, soaked in Masson A overnight and rinsed with tap water. Masson B and Masson C were prepared into Masson solution according to the ratio of 1:1. Then the sections were stained with Masson solution for 1 min, rinsed with tap water, differentiated with 1% hydrochloric acid alcohol and rinsed with tap water. After soaking in Masson D for 6 min, Masson E for 1 min and Masson F for 2–30 s successively, the slices were rinsed with 1% glacial acetic acid, dehydrated with two cups of anhydrous ethanol, soaked in Xylene for 5 min, sealed with neutral gum and imaged. 

### 2.12. Statistical Analysis

Statistical comparisons for more than three groups were analyzed by the one-way ANOVA analysis which was followed by the Tukey’s multiple comparison test using GraphPad Prism 8 software (GraphPad Prism 8.0.2, San Diego, CA, USA). *p* < 0.05 was considered statistically significant.

## 3. Results and Discussion

### 3.1. Fabrication and Characterization of Nanoparticles

SST was encapsulated in the PLGA nanoparticles via a facile nanoprecipitation strategy. To optimize the fabrication of SST-loaded nanoparticles, the influence of SST/PLGA weight ratios to the formation of nanoparticles was investigated. Obvious aggregates were formed above weight ratios of 1:20, whereas almost no aggregation was found at weight ratios of 1:50 and 1:100 (Table S1, Figure S1). Since higher input of cargos generally results in higher loading content [59,60], nanoparticles fabricated at 1:50 of SST/PLGA were utilized for following experiments. The UV-VIS spectrum of SST loaded PLGA nanoparticles (P-SST) showed a similar absorbance peak to free SST peptide at 284 nm, which did not appear in the spectrum of empty PLGA nanoparticles (P) (Figure S2), demonstrating the successful loading of SST. The encapsulation efficiency (EE) and loading efficiency (LE) of the peptide in P-SST were 73.68 ± 3.56% and 1.47 ± 0.07%, respectively. 

**Figure 2:**
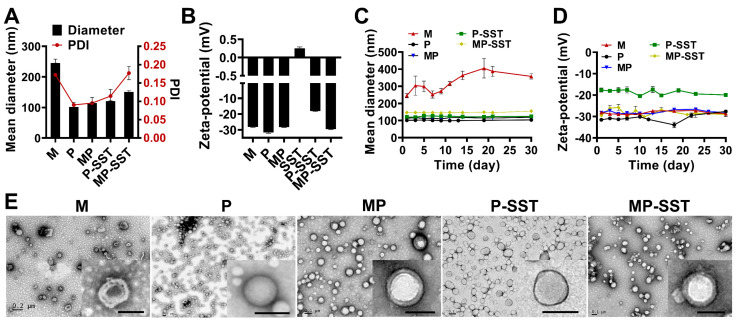
Characterization of nanoparticles. (**A**) The mean diameters and (**B**) zeta potentials of different nanoparticles and their long-term stabilities of (**C**) diameters and (**D**) zeta potentials (mean ± SD, *n* = 3). (**E**) The TEM images showed the morphology of nanoparticles (scale bar = 100 nm in bottom-right magnified images).

After the fabrication of P-SST, the macrophage membranes were utilized to camouflage the surfaces of nanoparticles. As shown in Figure 2A, the coating of membrane increased the diameters of nanoparticles from 101 ± 2 nm in P and 121 ± 1 nm in P-SST to 115 ± 1 nm in membrane-coated nanoparticles (MP) and 151 ± 4 nm in MP-SST, respectively. With a positive zeta potential, SST (0.25 ± 0.04 mV) increased the zeta potentials of the nanoparticles from −31.6 ± 0.6 mV in P to −18.1 ± 0.3 mV in P-SST. Under the coverage of the membrane, the MP and MP-SST showed similar zeta potentials to the membrane vesicles (M) (Figure 2B), indicating the transfer of the physicochemical properties of membrane materials onto the polymer nanoparticles. Different from the size increment of membrane vesicles under the storage in water for 1 month at 4 °C, the polymer-containing nanoparticles exhibited steady sizes and zeta potentials (Figure 2C,D), which demonstrated their stability. A hollow structure was shown in the TEM image of the membrane vesicles, while the membrane-coated nanoparticles showed the core-shell structure compared to the bare P or P-SST, directly proving the coating of cell membranes to the surfaces of nanoparticles (Figure 2E). The final MP-SST nanoparticles showed a two-phase SST release profile in which a first burst release was found within 24 h (about 50%) and followed by a sustained release, corresponding to the previously reported two-phase release profile of drugs in the membrane-coated PLGA nanoparticles [61,62]. 

### 3.2. Biocompatibility and Cellular Uptake of Nanoparticles In Vitro

**Figure 3:**
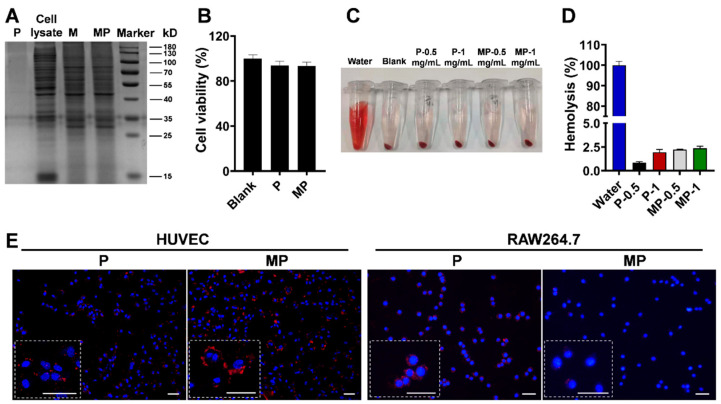
Biocompatibility and targeting ability of nanoparticles in vitro. (**A**) The SDS-PAGE electrophoresis analysis showed the protein profiles of macrophage cell lysate, membrane vesicles (M) and membrane-coated nanoparticles (MP). The bare PLGA nanoparticles (P) were used as a negative control. (**B**) Cell viability of HUVECs after incubation with P and MP (mean ± SD, *n* = 6). (**C**) The images of the centrifuged solutions containing erythrocytes after the incubation with P and MP nanoparticles and (**D**) the quantification of the absorbance intensities of the supernatants. The solutions of erythrocytes treated with pure water were utilized as positive control. (**E**) The fluorescent microscopic images showed the cellular uptake efficiencies of activated HUVECs and RAW264.7 macrophages after the incubation of nanoparticles labeled with DiI (Scale bar = 20 μm).

Given the importance of the proteins on the membrane to the nanoparticle properties, including the biocompatibility and targeting ability, the SDS-PAGE electrophoresis analysis was performed to investigate the protein components of the nanoparticles. The consistent protein profiles of MP with M, which were also found in the profiles of macrophage cell lysate, indicated the preservation and transfer of proteins from macrophage membranes to nanoparticles (Figure 3A). After incubation with the P and MP, the endothelial cells maintained their normal cell viabilities, which suggested the non-cytotoxicity of the nanoparticles (Figure 3B). Further analysis in the blood ex vivo showed that the hemolysis levels caused by all nanoparticles were below 5% (Figure 3C,D), demonstrating the biocompatibility of nanoparticles in circulation. It was found that the endothelial cells stimulated by TNF-α absorbed more MP than P, while the macrophages ingested more P than MP after 30 min of incubation (Figure 3E), suggesting the ability of MP to target the activated endothelial cells and escape the clearance by macrophages. All of that demonstrated the biosurface of the macrophage membrane enabled the MP with biocompatibility for administration and potential capability to accumulate in the inflammation-related sites in vivo.

### 3.3. Repair of Chronic Pancreatitis by Nanoparticles

**Figure 4:**
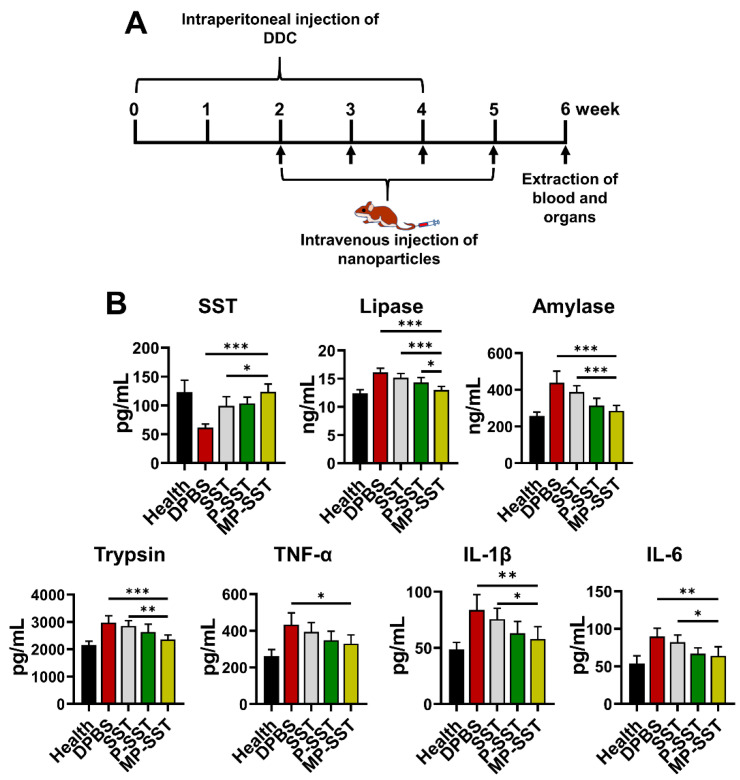
The modulation of chronic pancreatitis-related factors in mice. (**A**) The experimental design of investigation on the therapeutic effects of nanoparticles for the treatment of chronic pancreatitis in mice. (**B**) The protein contents of SST and chronic pancreatitis-related factors in the serum. All groups were compared to the MP-SST group. (Mean ± SD, *n* = 5~7; *, *p* < 0.05; **, *p* < 0.01; ***, *p* < 0.001.)

Chronic pancreatitis is associated with pancreatic atrophy, inflammation, fibrosis, exocrine dysfunction and endocrine disorder [63]. To investigate the regulation of these factors, nanoparticles were intravenously injected to mice with chronic pancreatitis induced by the diethyldithiocarbamate (DDC) [57,58]. In clinical management for pancreatitis, SST and some of its analogues are usually administered three times/day for successive days due to their short half-life [8]. Here, the treatments were administered once a week for four times to show the capability of nanoparticles in prolonging the therapeutic effects of SST. One week after the final administration, the blood and organs were extracted for analysis (Figure 4A). All SST-related treatments increased the content of SST in serum but the MP-SST enhanced the level to the highest extent compared to other treatment groups, demonstrating the efficiency of MP-SST to maintain the SST level as high as that in healthy mice. Owing to the recovery of SST content, the MP-SST showed the greatest ability to downregulate the chronic pancreatitis-related factors, including lipase, amylase, trypsin, TNF-α, IL-1β and IL-6 (Figure 4B). However, SST in its free form had a very modest and the lowest capability to regulate these factors, which may result from the fast degradation and short half-life of this peptide in vivo. All that suggested the capability of the membrane-coated nanoparticles to increase the content of SST to facilitate its regulatory functions in serum. 

**Figure 5:**
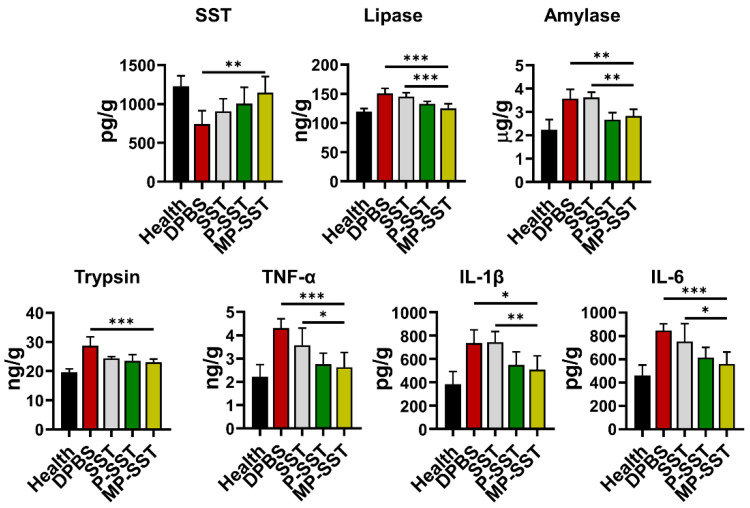
The protein contents of SST and chronic pancreatitis-related factors in the pancreas tissue. All groups were compared to the MP-SST group. (Mean ± SD, *n* = 5~7; *, *p* < 0.05; **, *p* < 0.01; ***, *p* < 0.001.)

In the pancreas, the P-SST also increased the content of SST compared to the administration of free SST, which may result from the attenuated degradation of the peptide when encapsulated in PLGA nanoparticles. However, SST content was further increased by MP-SST compared to the P-SST, demonstrating the efficacy of macrophage membrane coating to enhance the accumulation of SST in the injured pancreas (Figure 5). The increment of SST by the MP-SST resulted in the highest degree to downregulate the protein expressions of lipase, amylase, TNF-α, IL-1β and IL-6 to nearly normal levels in pancreas compared to other treatment groups (Figure 5), showing the superiority of membrane coating for nanoparticles to recover the levels of SST and chronic pancreatitis-related factors in pancreas. The PLGA nanoparticles coated with macrophage membrane have been administered via subcutaneous, intraperitoneal and intranasal administrations to target local bacterial infection, bacteremia and bacteria-loaded lung, respectively [48,64,65]. In this study, to deliver cargos to the pancreas, the nanoparticles were administered via intravenous injection, as MP nanoparticles loaded with drugs were intravenously injected for the treatment of acute pancreatitis previously [66]. After an intravenous administration, it was reported that the macrophage membrane surface may endow the nanoparticles with less plasma protein absorption and active trans-endothelium capability to pass through the vasculature in the inflamed sites [67,68]. However, whether these absorbed proteins affect the transportation mechanism of nanoparticles and if an active trans-endothelium or a passive (enhanced permeation and retention) EPR effect are involved in the transportation in this study may need further investigations. The study indicated that the increase in SST in pancreas by MP-SST can still have therapeutic effects though the liver was suggested to be the main organ to ingest most nanoparticles for elimination [34]. In the tissues, the PLGA in the MP-SST could be degraded via hydrolysis of esters to produce biologically inert glycolic and lactic acids and the membrane materials are innate natural materials from animals [69], suggesting the elimination of MP-SST from the body through common inherent metabolic pathways and the safety of the MP-SST. Since the therapeutic effect of SST for acute pancreatitis and the targeting ability of MP-based nanoparticles for acute pancreatitis have been demonstrated previously [5,66], the MP-SST may also have the potential to be applied for the treatment of acute pancreatitis.

**Figure 6:**
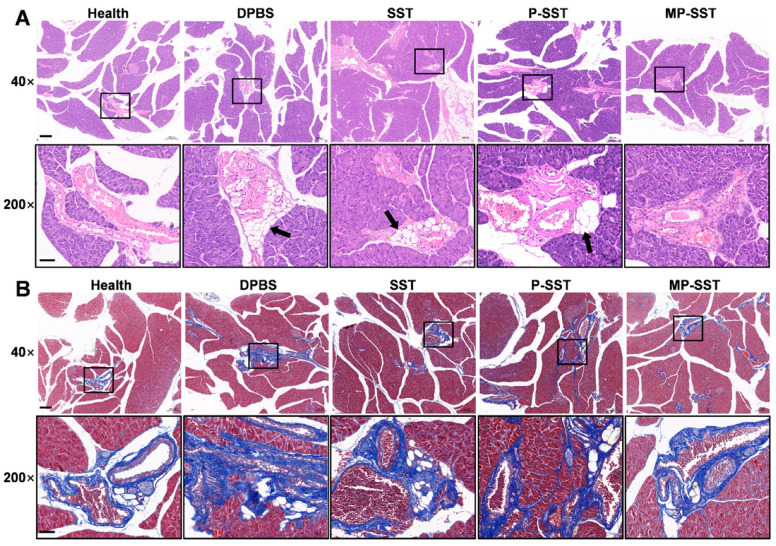
The histologic analysis of pancreas after different treatments. (**A**) Hematoxylin-eosin and (**B**) Masson’s trichrome staining of pancreas sections. Black arrows in (**A**) show the lipid infiltration/replacement around blood vessels. (Scale bars, 200 μm for 40× magnification and 50 μm for 200× magnification).

The mechanisms and clinical pathologic changes of chronic pancreatitis are quite complicated and involve activation of pancreatic enzymes, overwhelming of inflammatory cytokines, necrosis and atrophy of pancreatic acinar cells, infiltration of inflammatory cells, pancreatic fibrosis and lipid replacement [70]. Animal models with chronic pancreatitis can be created through different strategies, but whether these models produce all the features of human chronic pancreatitis remains unclear [71,72]. In this study, DDC, a superoxide dismutase (SOD) inhibitor, was used to induce oxidative stress and fibrosis in pancreas but not in the liver and kidney, which ultimately resulted in chronic pancreatitis [57,73]. At the sixth week post induction, the adipocytes were found to be present in the area between pancreatic lobules with particular accumulations around blood vessels (Figure 6A), which was in correspondence with the characteristics found in hamster and human [74,75], indicating the translational significance of this model in some degree. Compared to the treatment of DPBS and free SST, the administration of SST-loaded nanoparticles decreased the adipocyte accumulation/lipid replacement around blood vessels, whereas the MP-SST showed the greatest reduction (Figure 6A). The histologic analysis via Masson’s trichrome staining also showed the lowest lipid accumulation in the vascular periphery by the MP-SST treatment, which, meanwhile, seemed to reduce the fibrosis around these blood vessels (Figure 6B). It demonstrated the efficacy of macrophage membrane coating for MP-SST to inhibit the aggravation of chronic pancreatitis at the histologic level. The properties of the membrane biosurface and the polymer may all contributed to the final improvement of the overall therapeutic effects of MP-SST. 

## 4. Conclusions

In summary, a polymer-supported and biomimetic membrane surface-coated nanoparticulate platform was developed for therapeutic delivery of SST peptide to alleviate the severity of chronic pancreatitis. The nanoparticles were biocompatible and targetable to activated endothelial cells in vitro. Upon the recovery of SST content in serum and pancreas, the MP-SST reduced the expression of chronic pancreatitis-related factors and the histologic damages in pancreas to the greatest extent, demonstrating the superiority of integrating the synthetic polymer with natural biomembranes in the fabrication of nanoparticles to facilitate the functions of peptides. Utilizing similar design principles, this hybrid nanoplatform also holds promise for further application in the delivery of other peptides for the therapy of other inflammation-related diseases. Given MP-SST as an example, the study would inspire more advanced and intelligent biomimetic systems for peptide delivery to promote the translational process of peptides as drugs.

## Data Availability

The data presented in this study are available on request from the corresponding author.

## Supplementary Materials

The following supporting information can be downloaded at: https://www.mdpi.com/article/10.3390/pharmaceutics14112341/s1, Table S1: Diameters of nanoparticles formed at different weight ratios of SST/PLGA; Figure S1: Fabrication of nanoparticles under different weight ratios of SST/PLGA; Figure S2: UV-VIS spectrum of PLGA nanoparticles, SST peptide and P-SST dissolved in DMSO; Figure S3. The release profile of SST from the MP-SST nanoparticles.
