# Supplementary material for: A Macrophage Membrane–Polymer Hybrid Biomimetic Nanoplatform for Therapeutic Delivery of Somatostatin Peptide to Chronic Pancreatitis
Source: Pharmaceutics. 2022 Oct 30;14(11):2341. doi: 10.3390/pharmaceutics14112341 (PMC9698601; doi:10.3390/pharmaceutics14112341)
Supplement: Supplementary file 1 [file pharmaceutics-14-02341-s001.zip › pharmaceutics-1952414-supplementary.pdf]

Supplementary material

Table S1. Diameters of nanoparticles formed at different weight ratios of SST/PLGA.

DMSO/Water (V/V)	SST/PLGA (W/W)	Aggregates	Diameter (nm)	PDI
1:10	1:10	+++	177.10±2.10	0.07±0.02
1:10	1:20	+	150.32±1.01	0.11±0.01
**1:10**	**1:50**	**–**	**128.60±0.38**	**0.08±0.02**
1:10	1:100	–	117.96±1.49	0.15±0.03

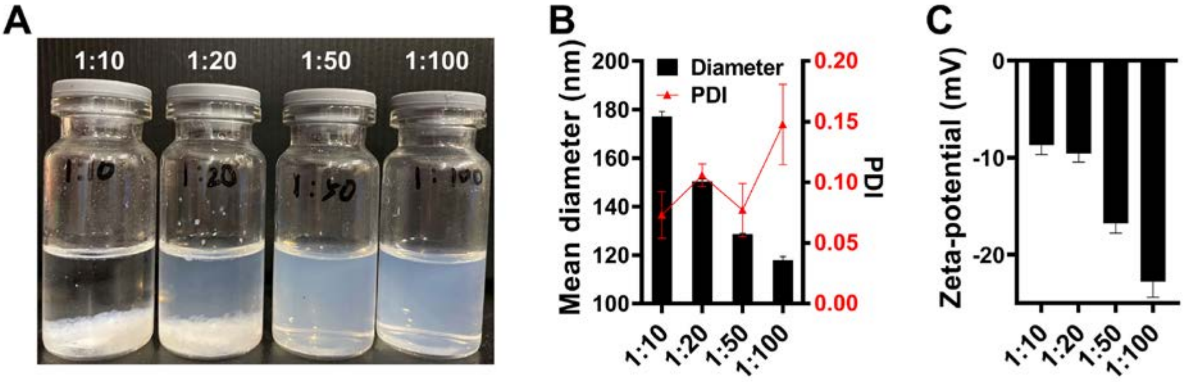

Figure S1. Fabrication of nanoparticles under different weight ratios of SST/PLGA. (A) The image of nanoparticle solutions fabricated under different weight ratios of SST/PLGA. Note the precipitation of nanoparticles at the bottom of vials under the conditions of 1:10 and 1:20 SST/PLGA weight ratios. (B) The diameters and (C) zeta-potentials of nanoparticles in the supernatant were increased by the increment of SST/PLGA weight ratios. (Mean ± SD, n=3).

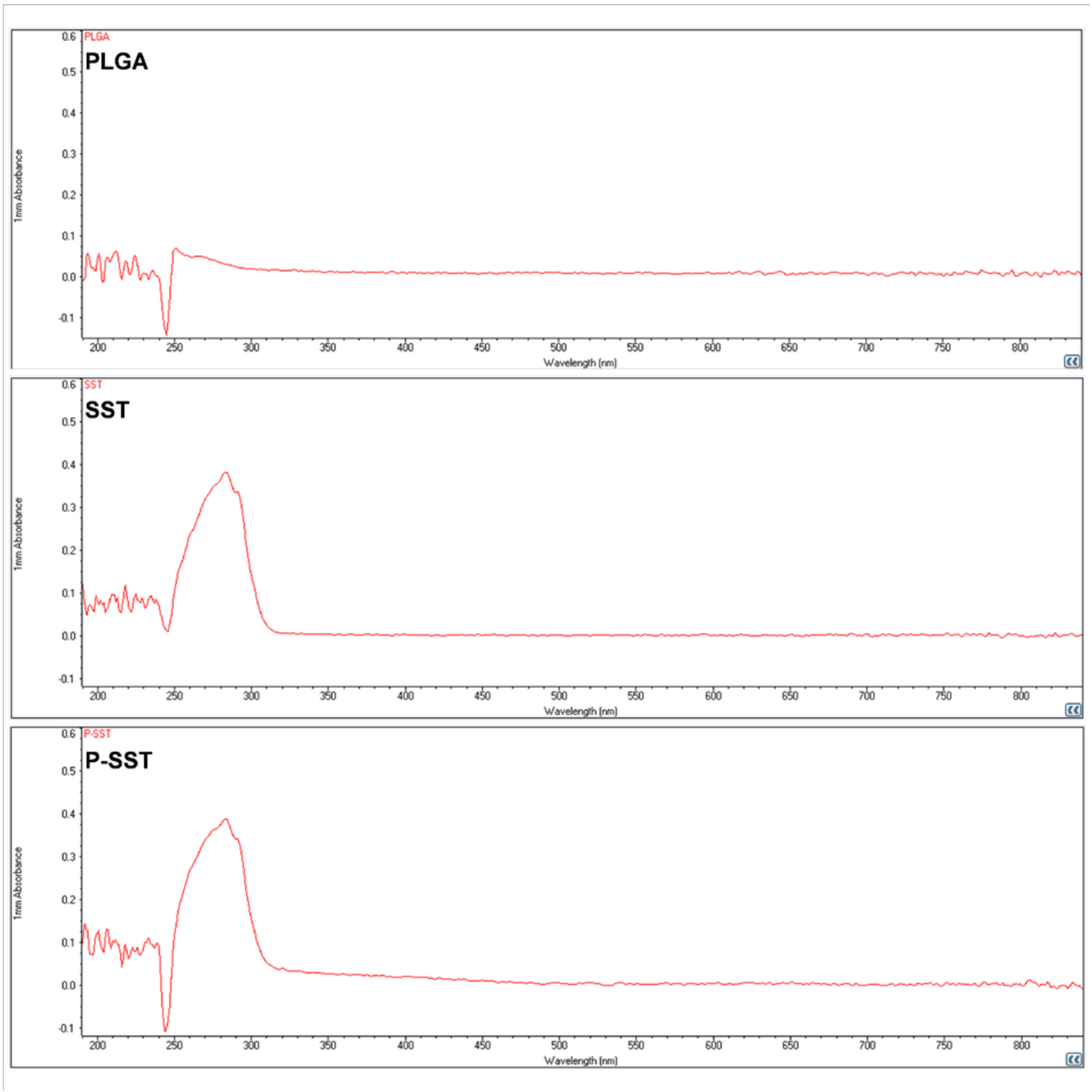

Figure S2. UV-VIS spectrum of PLGA nanoparticles, SST peptide and P-SST dissolved in DMSO. The spectrum of P-SST and SST showed a peak at 284 nm which was lack in the empty PLGA nanoparticles, demonstrating the successful encapsulation of SST in the P-SST nanoparticles.

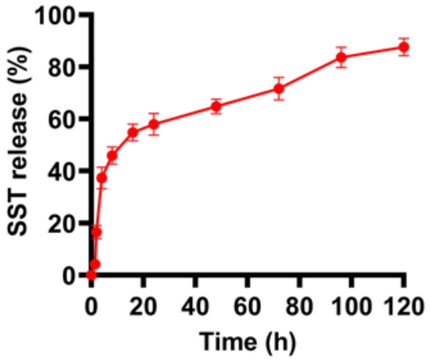

Figure S3. The release profile of SST from the MP-SST nanoparticles. (Mean ± SD, n=3).
